# *Scutellista caerulea* (Fonscolombe, 1832) (Hymenoptera: Pteromalidae), new to New Zealand for the second time!

**DOI:** 10.3897/BDJ.1.e959

**Published:** 2013-09-16

**Authors:** Stephen E. Thorpe

**Affiliations:** † School of Biological Sciences (Tamaki Campus), University of Auckland, Auckland, New Zealand

**Keywords:** *Scutellista caerulea*, Hymenoptera, Pteromalidae, New Zealand, Auckland, biological control, scale insects, NZOR

## Abstract

In 1921, *Scutellista caerulea* was imported and released in Nelson, New Zealand, for the biological control of pest scale insects. It was thought to have failed to establish, and is therefore currently considered to be absent from the New Zealand fauna. On 17 April 2013, a live specimen was captured in the wild in Auckland.

## Introduction

**Figure 1.**
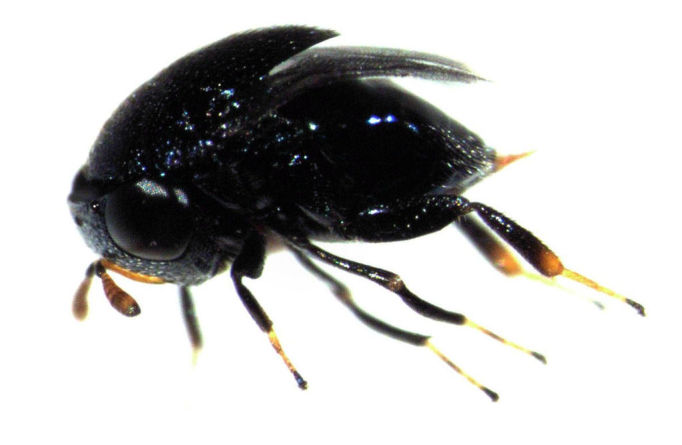
*Scutellista caerulea*, adult female in lateral view (length about 2 mm)

In 1921, *Scutellista caerulea* (Fonscolombe, 1832) was released in Nelson, New Zealand, for biological control of pest scale insects, but apparently failed to establish (Dumbleton 1936, as *Scutellista cyanea*). It is not considered to be present in New Zealand, and is not listed on the New Zealand Organisms Register (NZOR). However, on 17 April 2013, I captured a single live female in the wild, in the grounds of the Tamaki Campus of the University of Auckland (see Fig. 1). The species is so distinctive that it cannot be confused with any other, even within the genus *Scutellista*.

## Taxon treatments

### Scutellista caerulea

(Fonscolombe, 1832)

Scutellista cyanea Motschulsky, 1859

#### Materials

- **Type status:** Other material. **Occurrence:** recordedBy: Stephen Thorpe; individualCount: 1; sex: female; **Location:** country: New Zealand; verbatimLocality: Tamaki Campus of University of Auckland; verbatimLatitude: 36.8816119078S; verbatimLongitude: 174.8531936109E; **Event:** eventDate: 17 April 2013; **Record Level:** institutionCode: Auckland Museum

#### Description

The specimen was identified using Bouček (1988), and references listed therein. I recommend that *Scutellista caerulea* be added to the New Zealand Organisms Register (NZOR) as exotic, present in the wild. It is unclear if the new specimen represents a descendant of the original stock released in Nelson in 1921, or a new incursion from overseas. The balance of evidence favours the latter hypothesis, since Auckland is far from Nelson, and the species has not been seen in N.Z. for nearly a century.

## Supplementary Material

XML Treatment for Scutellista caerulea

## References

[B141162] Bouček Z (1988). Australasian Chalcidoidea (Hymenoptera). A biosystematic revision of genera of fourteen families, with a reclassification of species.

[B141171] Dumbleton L. J. (1936). The biological control of fruit pests in New Zealand. New Zealand journal of science and technology.

